# *Ocimum tenuiflorum* Essential Oil as a Safe Tool to Prevent *Neocosmospora keratoplastica* Fungus in *Adenium obesum* Ornamental Plant

**DOI:** 10.3390/plants15152332

**Published:** 2026-07-29

**Authors:** Vitória Beatriz Silva, Adauto A. Silva Júnior, Dalmarcia de Souza C. Mourão, Paulo Ricardo de S. Fernandes, Rosilene da Costa P. de Carvalho, Ana G. Amaral, Marcos Paz S. Câmara, Eugênio E. Oliveira, Raimundo W. de Sousa Aguiar, Ildon R. do Nascimento, Marcos V. Giongo, Marcos G. da Silva, Eduardo Valarezo, Luis O. Viteri, Gil R. dos Santos

**Affiliations:** 1 Departamento de Fitopatologia, Universidade Federal do Tocantins, Gurupi 77402-970, TO, Brazil; vitoria.beatriz@mail.uft.edu.br (V.B.S.); dalmarciaadm@uft.edu.br (D.d.S.C.M.); pauloricardosena@mail.uft.edu.br (P.R.d.S.F.); rosilene.porto@mail.uft.edu.br (R.d.C.P.d.C.); 2 Programa de Pós-Graduação em Biotecnologia, Universidade Federal do Tocantins, Gurupi 77402-970, TO, Brazil; eugenio@ufv.br (E.E.O.); rwsa@uft.edu.br (R.W.d.S.A.); gontijobio@uft.edu.br (M.G.d.S.); luis.viteri@uft.edu.br (L.O.V.); 3 Departamento de Ciências Biológicas, Universidade Estadual de Feira de Santana (UEFS), Novo Horizonte 44036-900, BA, Brazil; adauto@mail.uft.edu.br; 4 Instituto Federal de Educação, Ciência e Tecnologia do Sertão Pernambucano, Campus Floresta, Floresta 56400-000, PE, Brazil; gabriele.160713@gmail.com; 5 Departamento de Agronomia, Universidade Federal Rural de Pernambuco, Recife 52171-900, PE, Brazil; marcos.camara@ufrpe.br; 6 Programa de Pós-Graduação em Produção Vegetal, Universidade Federal do Tocantins, Gurupi 77402-970, TO, Brazil; ildon@uft.edu.br (I.R.d.N.); giongo@uft.edu.br (M.V.G.); 7 Departamento de Química y Producción, Universidad Técnica Particular de Loja, Loja 110107, Ecuador; bevalarezo@utpl.edu.ec

**Keywords:** natural antifungal agents, plant disease management, ornamental plant pathology, antifungal activity, in silico antifungal target proteins

## Abstract

*Adenium obesum* (Forssk.) Roem. & Schult (desert rose) is one of the most popular ornamental species cultivated in residential gardens and as a potted plant for balconies and verandas in Brazil. However, *Neocosmospora keratoplastica* has recently been identified as one of the causal agents of rot disease in this species. Therefore, this study evaluated the antifungal potential of *Ocimum tenuiflorum* L. essential oil (EO) as a preventive and curative treatment against this pathogen, while assessing its phytotoxicity and selectivity toward beneficial fungi. The EO was obtained by hydrodistillation and chemically characterized. In vitro mycelial growth inhibition assays, in vivo evaluations on *A. obesum* seedlings, and selectivity tests against *Trichoderma asperellum* were performed. In addition, in silico molecular docking analyses were conducted to investigate the interaction of the major EO constituents with fungal target proteins. Eugenol (47.04%) and 1,8-cineole (29.47%) were identified as the predominant compounds in the EO. Mycelial growth inhibition reached a maximum of 59% at 100 μL mL^−1^, but strong phytotoxicity was evident; while a concentration of 50 μL mL^−1^ yielded an 18% inhibition rate representing an approximately three-fold greater effect than that of thiophanate methyl (7.68%) at the recommended dose, with a slight phytotoxicity. The lowest Area Under the Disease Progress Curve (AUDPC) was recorded when the EO was applied preventively, and intermediate concentrations were selective toward *T. asperellum*. Molecular docking analyses indicated that eugenol and 1,8-cineole exhibited greater affinity for CYP51A than for α/β-tubulin, whereas dehydroaromadendrane and α-copaene showed the strongest interactions with these target proteins. Overall, *O. tenuiflorum* EO exhibits promising potential as a preventive treatment against *N. keratoplastica* in desert rose, while preserving beneficial fungi at moderate concentrations.

## 1. Introduction

*Adenium obesum* (Forssk.) Roem. & Schult, popularly known as the desert rose, belongs to the Apocynaceae family and is an Angiosperm native to Sub-Saharan Africa and the Arabian Peninsula. Currently, it is cultivated under a wide range of environmental conditions, from humid to arid regions, and is therefore widely distributed in diverse settings. In Brazil the desert rose has become particularly prominent in the northeastern region due to its adaptation to conditions of extreme heat [1]. In addition to its compacted size, which facilitates its cultivation in medium-sized pots, this specie is characterized by the sculptural caudex and root system, tolerance to water stress, and the wide diversity of flower colors among cultivars. These characteristics make *A. obesum* one of the most popular ornamental species in residential gardens and one of the most desirable potted plants for balconies and verandas, particularly in areas where green space is limited. The benefits of cultivating ornamental plants extend beyond their aesthetic value, as a recently study by Bayazit Solak and Kisakurek [2], demonstrated that growing plants on balconies and verandas has positive effects on people’s physical and mental health. However, for ornamental plants to express their full aesthetic potential, they must remain free from pest and disease infestations, and, like all plant species, *A. obesum* is susceptible to attacks by a variety of pests and pathogens.

Fungal disease are among the main phytosanitary challenges in ornamental plant production due to the ability of these pathogens to persist in the soil and infect a wide range of hosts [3]. Several fungal genera are associated with ornamental plants, including *Colletotrichum* [4,5], *Alternaria* [6], *Fusarium* [4], *Phytophthora* [7], *Cercospora* [8], *Pestalotiopsis* [9] and *Neocosmospora* [10]. Likewise, the desert rose has been reported as a host of several fungal pathogens, mainly species of *Colletotrichum*, *Alternaria and Aspergillus*, among others [11,12]. More recently Silva et al. [13] reported *Neocosmospora keratoplastica* as the causal agent of wilt and stem rot in *A. obesum* in Brazil, representing the first report worldwide. Infections caused by these pathogens compromise not only plant development and survival but also their aesthetic quality, an essential attribute that determines the commercial value of ornamental species.

Although *Neocosmospora keratoplastica* has been reported as the causal agent of wilt and root rot in members of the Cucurbitaceae [14], it has also recently been included among the priority fungal pathogens because of this opportunist nature and its ability to infect humans and animals, causing severe clinical manifestations [15,16]. This characteristic highlights the need for special attention to its management, particularly when the pathogen occurs in ornamental plants maintained in residential environments, where close and frequent human-plant contact may increase the risk of exposure. Therefore, the diagnosis and control of this pathogen in ornamental plants should be considered a priority. Although the management of these diseases relies largely on the use of synthetic fungicides, this practice may be problematic, especially in desert rose, which is commonly cultivated as a potted plant on balconies and verandas. Moreover, the continuous application of these compounds may result in environmental impacts, risks to non-target organisms, and the selection of resistant pathogen populations [17,18]. *Trichoderma asperellum*, a beneficial fungus commonly found in soil and plant root systems, acts as a natural biocontrol agent, promotes plant growth, improves soil health, and induces plant resistance. Therefore, *Trichoderma* species are commonly unable to work together in controlling fungal diseases because they are sensitive to synthetic fungicides [19,20]. Thus, the compatibility between any fungicides and the biological control agents, such as the fungus *Trichoderma*, should be evaluated prior to the potential use in a combined control agent. Essential oils have emerged as promising sources of bioactive metabolites, and, in general, these compounds exhibit low toxicity toward non-target organisms [21].

Essential oils consist of complex mixtures of volatile compounds, including monoterpenes, sesquiterpenes, and phenolic compounds, whose synergistic interactions may confer potential antifungal effects [22,23]. Additionally, these products have shown selectivity toward beneficial microorganisms [20] pollinators [24], natural enemies [21,25] and mammals [26], and more recently have been reported to induce plant defense responses against fungal pathogens [23,27]. Consequently, interest has increased in plant species with recognized bioactive potential, including *Ocimum tenuiflorum* L. (Tulsi). This species belongs to the Laminaceae family and has been widely used for numerous therapeutic purposes [28]; therefore, its use as a bioproduct for pathogen management in ornamental plants is expected to be safe for applicators. The essential oil of *O. tenuiflorum* is rich in eugenol and 1,8-cineole, compounds known for their antifungal properties against *Botryotinia fuckeliana*, *Rhizoctonia solani*, *Alternaria* spp. and *Verticillium dahlia* [29,30]. However, information regarding the activity of these compounds, and particularly of *O. tenuiflorum* essential oil, against the *Neocosmospera keratoplastica* in ornamental plants such as *Adenium obesum* remains limited. Therefore, the present study evaluated the potential use of *O. tenuiflorum* essential oil for the control of caudex rot caused by *N. keratoplastica*, considering the phytotoxicity, compatibility and possible effects on *Trichoderma asperellum* as non-target organisms. Additionally, in silico analyses using bioinformatic tools were performed to elucidate the potential target sites of the compounds present in this essential oil.

## 2. Results

### 2.1. Chemical Characterization of the Essential Oil of Ocimum tenuiflorum

**Figure 1:**
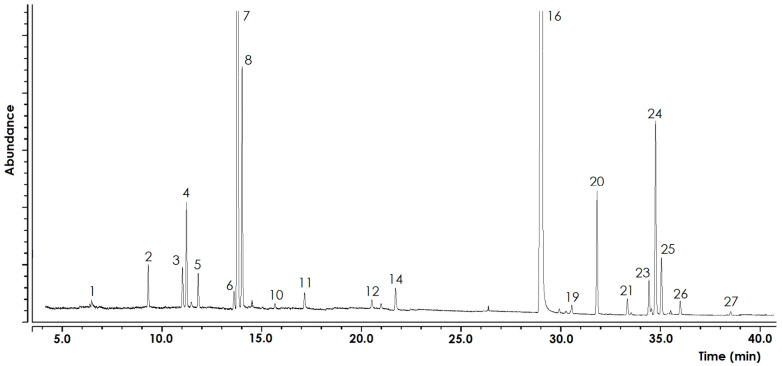
Representative chromatograms of the essential oil of *Ocimum tenuiflorum*; the numbers correspond to the compound numbers in Table 1.

The chemical characterization of the essential oil of *O. tenuiflorum* (Figure 1) revealed the presence of 27 identified compounds, representing 98.58% of the total oil composition. Eugenol (47.04%) and 1,8-cineole (29.47%) were the predominant constituents, together accounting for more than 75% of the total composition. The chemical profile consisted mainly of oxygenated monoterpenes (77.78%), followed by monoterpene hydrocarbons (10.59%) and sesquiterpene hydrocarbons (9.97%) (Table 1).

### 2.2. Antifungal Activity of Ocimum tenuiflorum Essential Oil Against Neocosmospora keratoplastica

The antifungal activity of *O. tenuiflorum* essential oil was concentration-dependent and did not completely inhibit mycelial growth at any of the concentrations tested. The concentrations evaluated in this study were insufficient to achieve complete control of *N. keratoplastica*, with the highest concentration resulting in only 59% inhibition of mycelial growth. However, from a concentration of 40 µL mL^−1^ onward, the essential oil exhibited greater inhibitory activity against mycelial growth than the synthetic fungicide (Table 2).

### 2.3. Effect of Ocimum tenuiflorum Essential Oil on the Phytotoxicity of Adenium obesum

**Figure 2:**
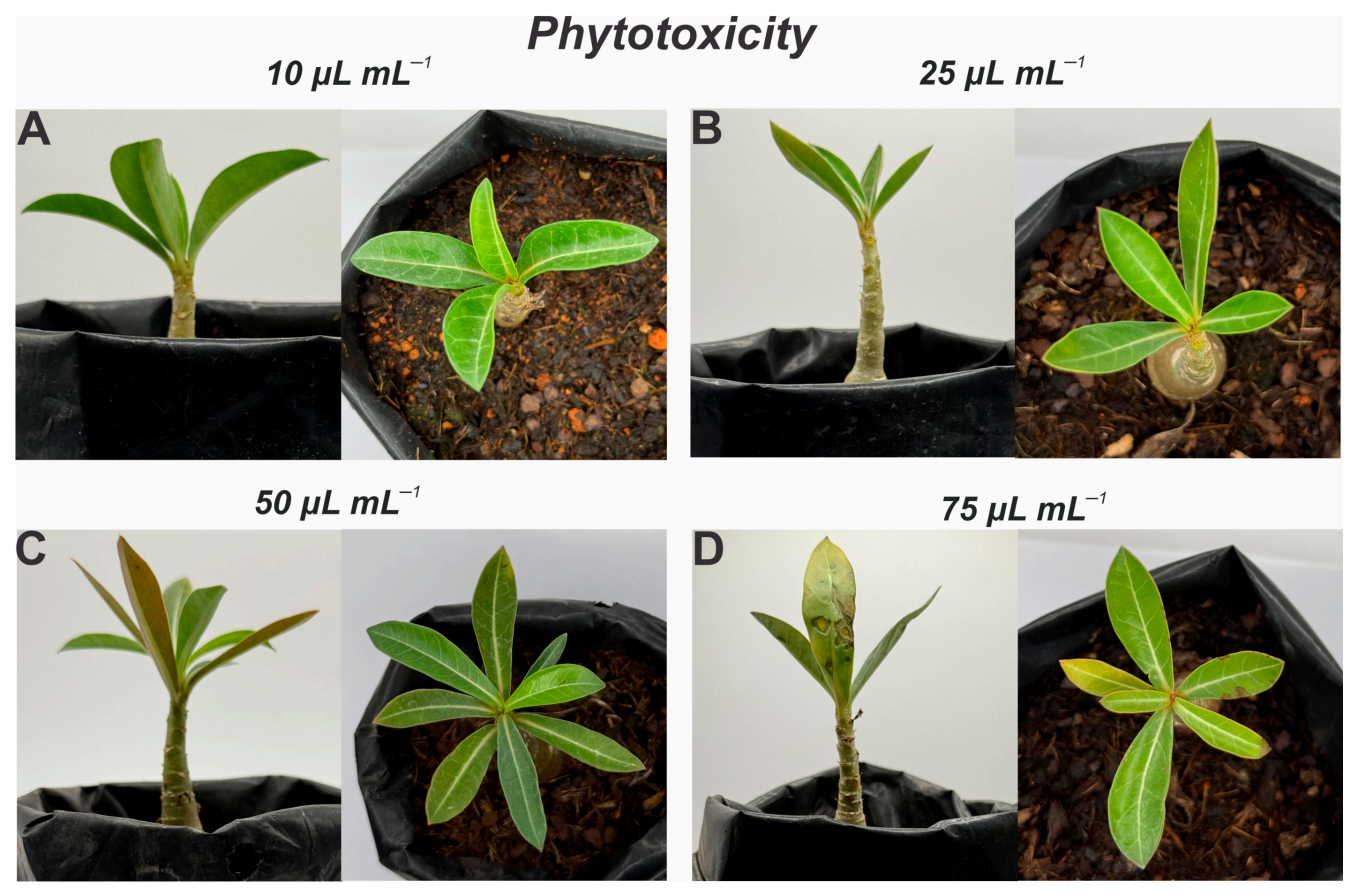
Phytotoxicity effects of different concentrations of *Ocimum tenuiflorum* essential oil (**A**–**D**) on *Adenium obesum* seedlings 24 h after application.

The phytotoxic effect of *O. tenuiflorum* essential oil on *A. obesum* was concentration-dependent. No phytotoxic effects were observed at the lowest concentrations tested (Figure 2A,B). However, at 50 µL mL^−1^, mild phytotoxicity was detected and was characterized by leaf chlorosis (Figure 2C). At the highest concentration, the effects were more severe, with the development of necrotic lesions associated with chlorosis (Figure 2D).

### 2.4. Effect of Ocimum tenuiflorum Essential Oil on the Preventive and Curative Control of Caudex Rot Caused by Neocosmospora keratoplastica

**Figure 3:**
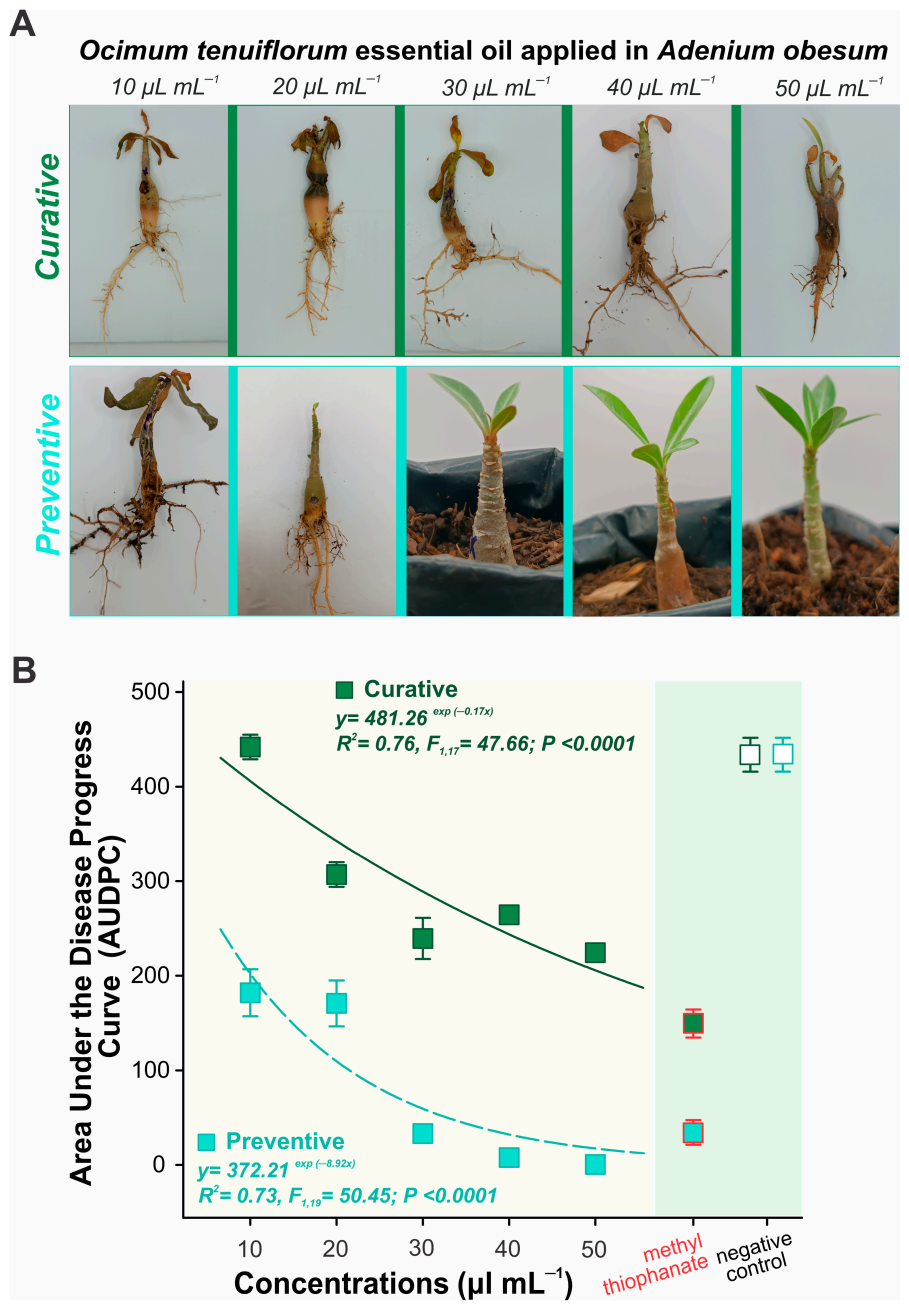
Area Under the Disease Progress Curve (AUDPC) of *Adenium obesum* seedlings evaluated 10 days after inoculation and subjected to preventive and curative treatments with different concentrations of *Ocimum tenuiflorum* essential oil (**A**,**B**). Symbols represent the mean (±SE) of five replicates and red and white color represent the mean (±SE) of chemical and negative control respectively.

Ten days after treatment application, a reduction in the Area Under the Disease Progress Curve (AUDPC) was observed in plants treated with *O. tenuiflorum* essential oil, and this effect was dependent on both the concentration and the timing of application (Figure 3A). When applied as a preventive treatment, the essential oil was more effective than when applied as a curative treatment at all concentrations tested. Moreover, the preventive application exhibited an effect similar to that of the chemical control at the three highest concentrations evaluated (Figure 3B). Likewise, a concentration-dependent effect was observed when the essential oil was applied as a curative treatment; however, its efficacy remained lower than that of the chemical control, even at the highest concentrations tested (Figure 3B). Greater control efficiency has been observed starting at a concentration of 30 µL mL^−1^, for both preventive and curative control.

### 2.5. Interactions Between Ocimum tenuiflorum Essential Oil and the Target Sites of Neocosmospora keratoplastica

#### 2.5.1. Homology Modeling of CYP51A, α/β-Tubulin and Validation of the Heme Cofactor

**Figure 4:**
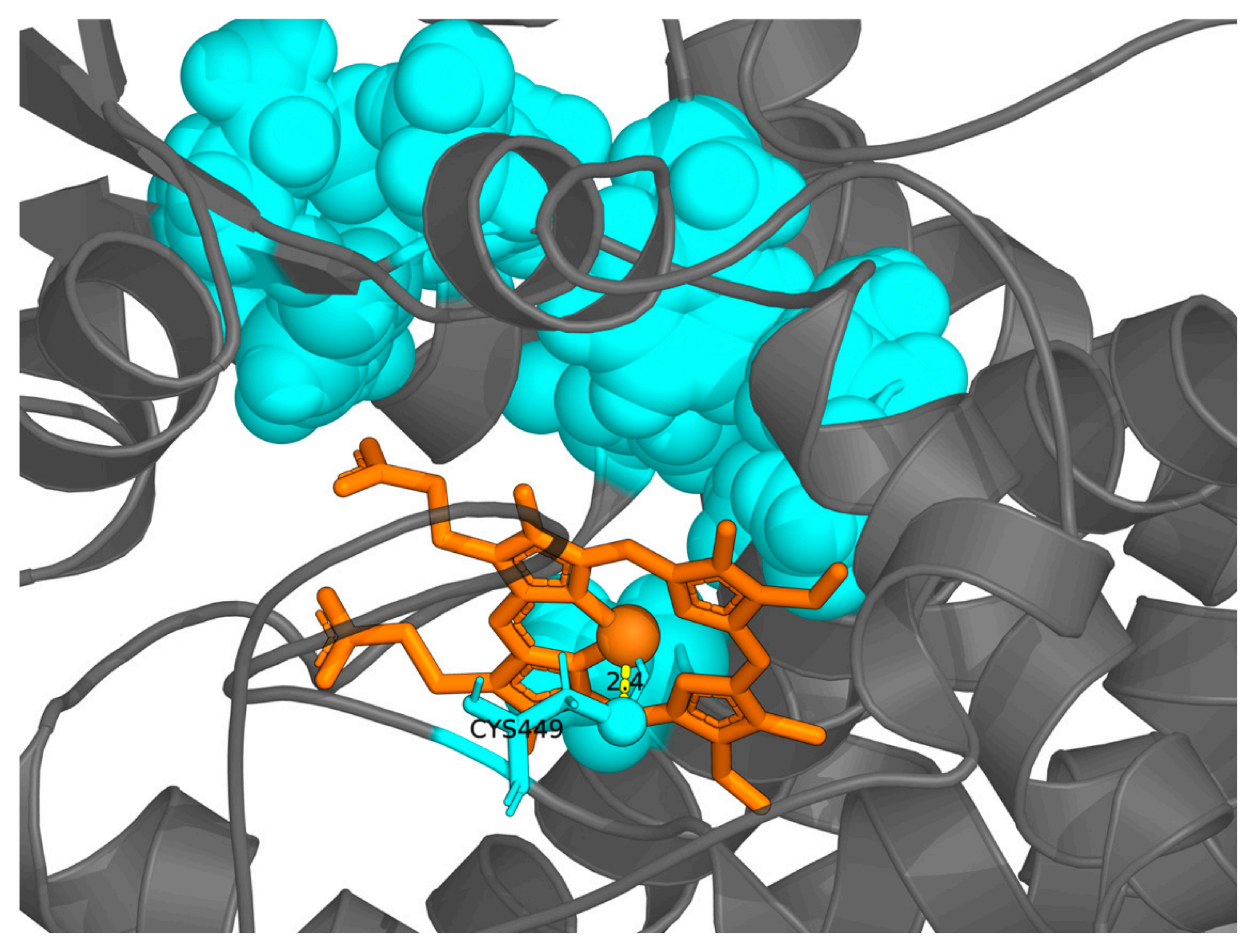
Homology model showing coordination of the heme group (orange) within the active site of the CYP51A protein (blue).

The structural model of CYP51A generated using AlphaFold2 exhibited high quality (pLDDT = 91.2; pTM = 0.903), revealing a helical catalytic core typical of cytochrome P450 enzymes and a well-defined active site (Figure S1A,B) [31,32]. Ramachandran analysis showed that 93.7% of the residues were located in favored regions (Figure S1C), whereas the Predilect Aligned Error (PAE) matrix indicated low structural uncertainty (Figure S1D). The heme cofactor was successfully incorporated using AlphaFill and exhibited an ideal planar geometry (RMSD = 0.020 Å), an Fe–S(Cys449) distance of 2.40 Å, and no steric clashes (Figure 4), thereby validating the model for subsequent computational studies.

The α/β-tubulin heterodimeric model demonstrated high reliability (pLDDT = 90.2; pTM = 0.907; ipTM = 0.911), with a well-defined CBS binding site at the α/β interface (Figure S2A,B). The PAE map revealed low uncertainty across both subunits and the interface, while Ramachandran analysis confirmed 93.7% of residues were located in favored regions, with no residues in disallowed regions, thereby validating the model for studies on fungal β-tubulin inhibition (Figure S2C,D).

#### 2.5.2. Molecular Docking Performance of the 27 Essential Oil Ligands and Synthetic Fungicides Against CYP51A and α/β-Tubulin

The docking performance of the 27 essential oil ligands against CYP51A and α/β-tubulin revealed a gradient of binding energies (ΔG). Overall, CYP51A exhibited more negative (ΔG) values, ranging from −3.89 Kcal/mol for (3E)-Hexenol to −8.69 kcal/mol for dehydroaromadendrane (Table 3). For α/β-tubulin, a similar trend was observed, although with less favorable binding energies, ranging from −3.17 kcal/mol for (3E)-Hexenol to −7.39 kcal/mol for α-Copaene (Table 3). Among the major compounds of the essential oil of *O. tenuiflorum*, eugenol and 1,8-cineole showed binding affinities of −5.10 and −5.88 Kcal/mol against CYP51A, respectively, while for *α/β-tubulina* the values were −4.16 Kcal/mol (eugenol) and −5.54 (1,8-cineole) (Table 3). When evaluated against CYP51A, the commercial azole inhibitors showed that itraconazole exhibited the highest binding affinity (ΔG = −10.00 ± 1.07 kcal/mol), whereas voriconazole and tebuconazole displayed similar values (−6.81 ± 0.22 and −6.87 ± 0.05 kcal/mol, respectively) (Table 3). RMSD values between poses (0.42–1.36 Å) and distances to the heme group (2.25–2.42 Å) indicated consistent convergence and appropriate positioning of the azoles within the CYP51A active site. Regarding α/β-Tubulin, benzimidazole and nocodazole exhibited binding affinities of −3.86 kcal/mol and +2.75 kcal/mol, respectively (Table 3). RMSD values between replicates were extremely low (0.00–0.06 Å), indicating consistent convergence and high conformational reproducibility across three replicates of 100 runs. Although Nocodazole showed a positive ΔG value, it still displayed low RMSD, suggesting convergence toward a consistent binding pose. In contrast, benzimidazole and carbendazim presented negative ΔG values.

#### 2.5.3. Molecular Docking of Dehydroaromadendrane, Eugenol, and 1,8-Cineole in CYP51A Active Site

**Figure 5:**
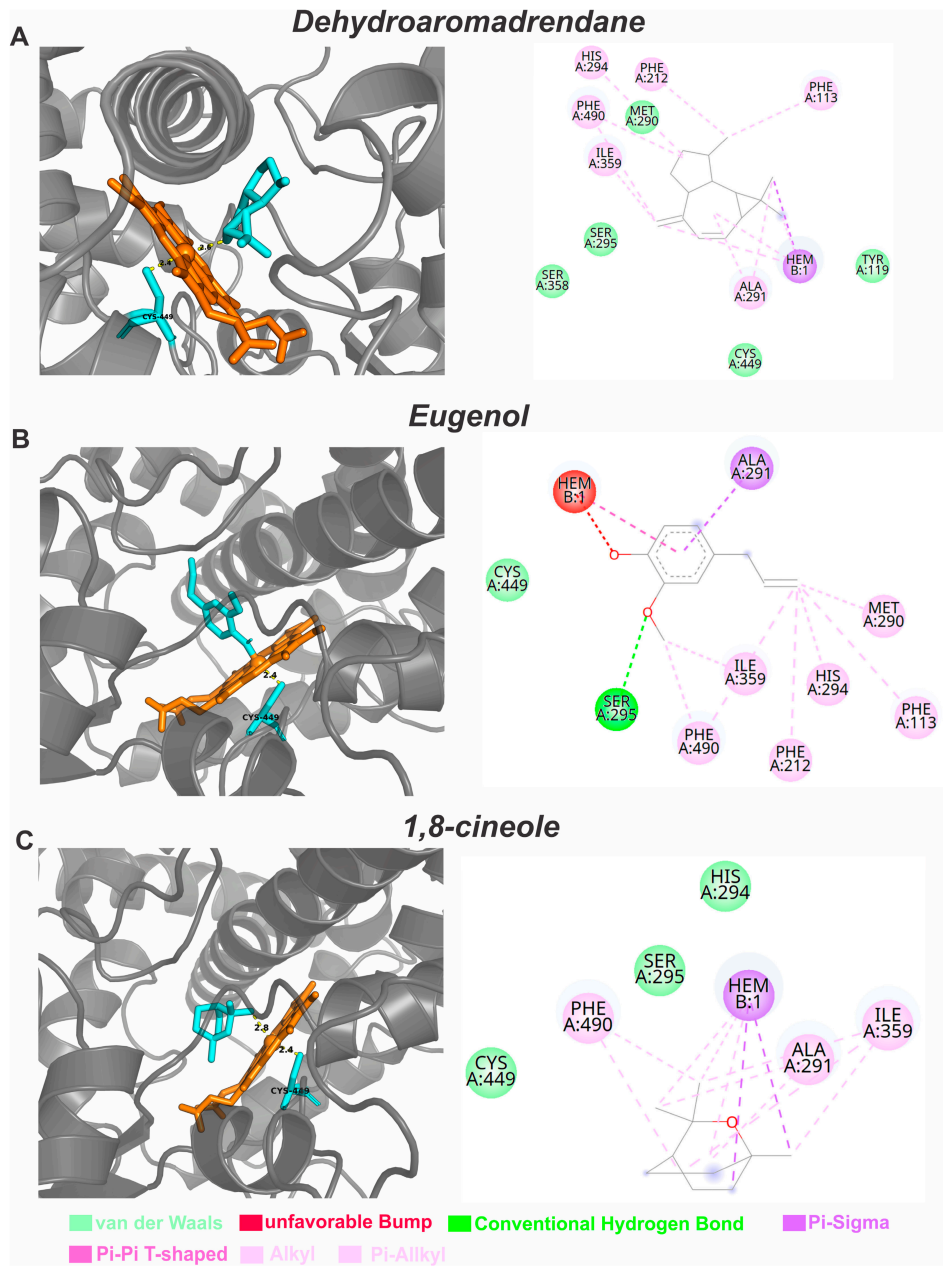
Three-dimensional models of the CYP51A complexes with dehydroaromadendrane (**A**), eugenol (**B**) and 1,8-cineole (**C**), showing the positioning of each ligand within the catalytic site (blue) relative to the heme group (orange). Interaction diagrams for each ligand within the active site are shown on the right side of each panel.

As shown previously, the sesquiterpene dehydroaromadendrane exhibited the most favorable ΔG in the CYP51A screening (−8.69 kcal/mol). Structural analysis revealed predominantly hydrophobic interactions with residues PHE212, MET290, and HIS294 (Figure 5A), with the compound aligning parallel to the heme plane at a distance of 2.6 Å (Figure 5A). Van der Waals, alkyl, and pi-alkyl contacts predominated, with no hydrogen bond formation. Among the major compounds, eugenol exhibited a ΔG of −5.10 kcal/mol, placing it among the least favorable ligands. Two-dimensional interaction analysis revealed hydrophobic contacts with residues PHE212, MET290, ALA291, and HIS294, as well as a hydrogen bond between the hydroxyl group and SER295 (Figure 5B). However, an unfavorable interaction was observed between the methoxy group and the heme cofactor (Figure 5B). The monoterpene 1,8-cineole exhibited a ΔG of −5.88 kcal/mol against CYP51A, corresponding to an intermediate binding affinity. Structural analysis revealed predominantly hydrophobic interactions with residues ALA291, HIS294, SER295, and the heme cofactor, with no hydrogen bond formation (Figure 5C). The compound was positioned close to the heme plane (2.6 Å), with its oxygen atom oriented toward the porphyrin ring (Figure 5C).

#### 2.5.4. Molecular Docking on the α-Copaene, Eugenol and 1,8-Cineole in α/β-Tubulin Heterodimer (Colchicine Binding Site)

**Figure 6:**
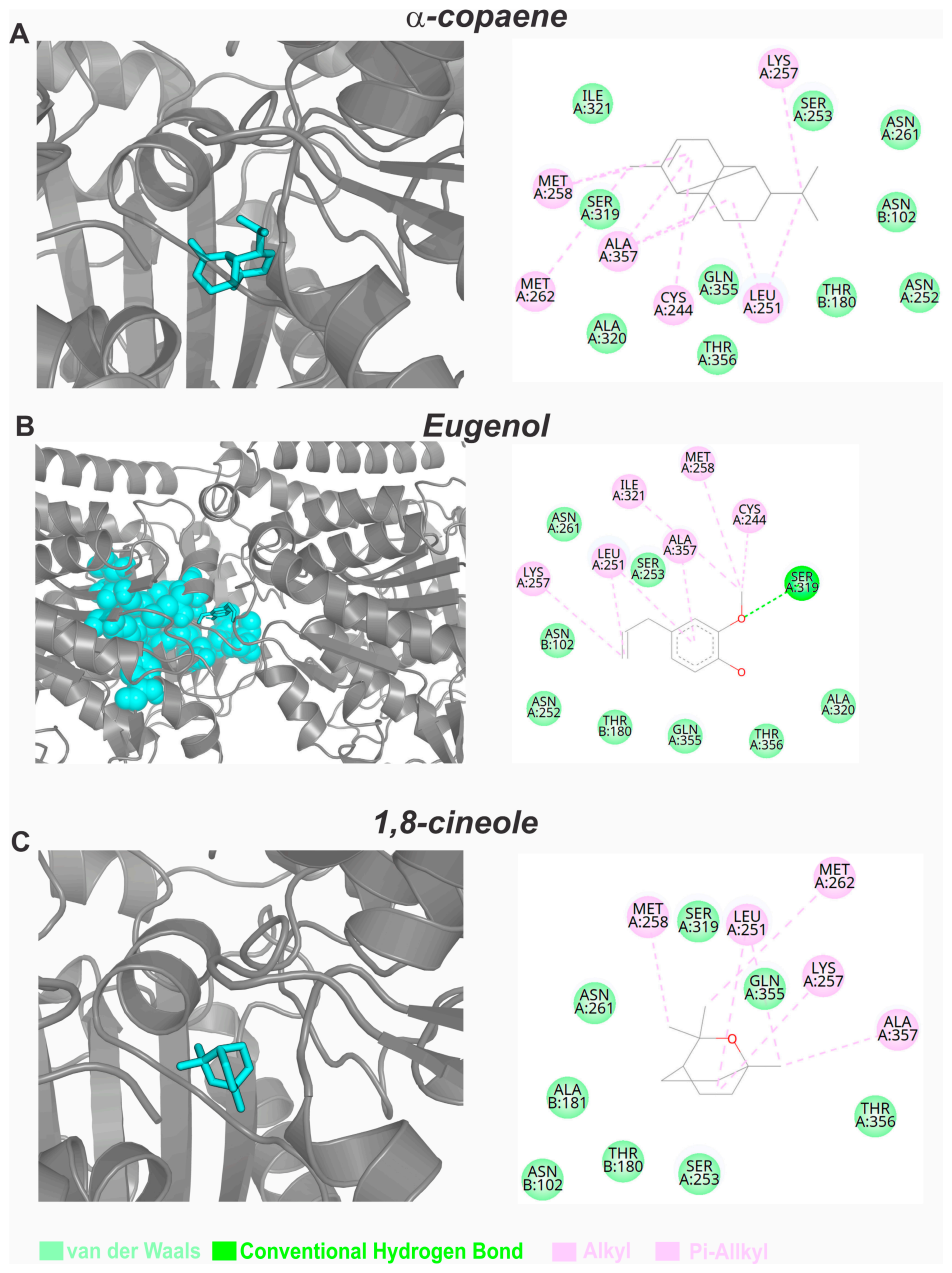
Three-dimensional models of the α/β-tubulin– α-Copaene (**A**), eugenol (**B**) and 1,8-cineole (**C**) complex showing the positioning of each ligand within the colchicine binding site (CBS) (blue). Interaction diagrams for each molecule within the active site are shown on the right side of each panel.

The sesquiterpene α-copaene exhibited the most favorable ΔG in the α/β-tubulin screening (−7.39 kcal/mol), accommodating itself within the Colchicine Binding Site (CBS) cavity. Two-dimensional interaction analysis indicated predominantly hydrophobic contacts with β-tubulin residues, including CYS244, LEU251, MET258, ILE321, and ALA357, as well as contributions from α-tubulin (ASN102 and THR180). Three-dimensional visualization revealed deep insertion of the ligand into the interfacial cavity, with an orientation favorable for hydrophobic interactions. Comparatively, α-copaene exhibited a more negative ΔG than benzimidazole (−4.15 kcal/mol) and carbendazim (−3.86 kcal/mol) (Figure 6A). Eugenol exhibited a ΔG of −4.16 kcal/mol against α/β-tubulin, placing it among the least favorable ligands, with affinity comparable to benzimidazole. Two-dimensional analysis revealed interactions with CBS residues CYS244, LEU251, MET258, ILE321, THR356, and ALA357 in β-tubulin, as well as hydrophobic contacts with ASN102 and THR180 of α-tubulin, and a hydrogen bond with SER319 (Figure 6B). Three-dimensional visualization showed eugenol positioned within the CBS, with its aromatic moiety aligned along the cavity walls, its allylic chain oriented toward the pocket opening, and its polar group directed toward SER319. The 1,8-cineole exhibited a ΔG of −5.54 kcal/mol against α/β-tubulin, corresponding to an intermediate binding affinity. Two-dimensional analysis revealed predominantly alkyl and pi-alkyl interactions with residues LEU251, MET258, SER319, THR356, ALA357, and chain B residues (THR180, ASN102, ALA181), with no hydrogen bond formation (Figure 6C). Three-dimensional visualization showed partial occupation of the CBS pocket, with an orientation parallel to the hydrophobic cavity walls (Figure 6C). The smaller molecular volume of 1,8-cineole compared to sesquiterpenes may account for its lower estimated binding affinity. 1,8-cineole established exclusively hydrophobic interactions with CBS residues, whereas eugenol, owing to its phenolic hydroxyl group, also formed a hydrogen bond. Nevertheless, 1,8-cineole exhibited a ΔG approximately 1.4 kcal/mol more favorable than eugenol.

### 2.6. Effect of Ocimum tenuiflorum Essential Oil on the Non-Target Organism Trichoderma asperellum

**Figure 7:**
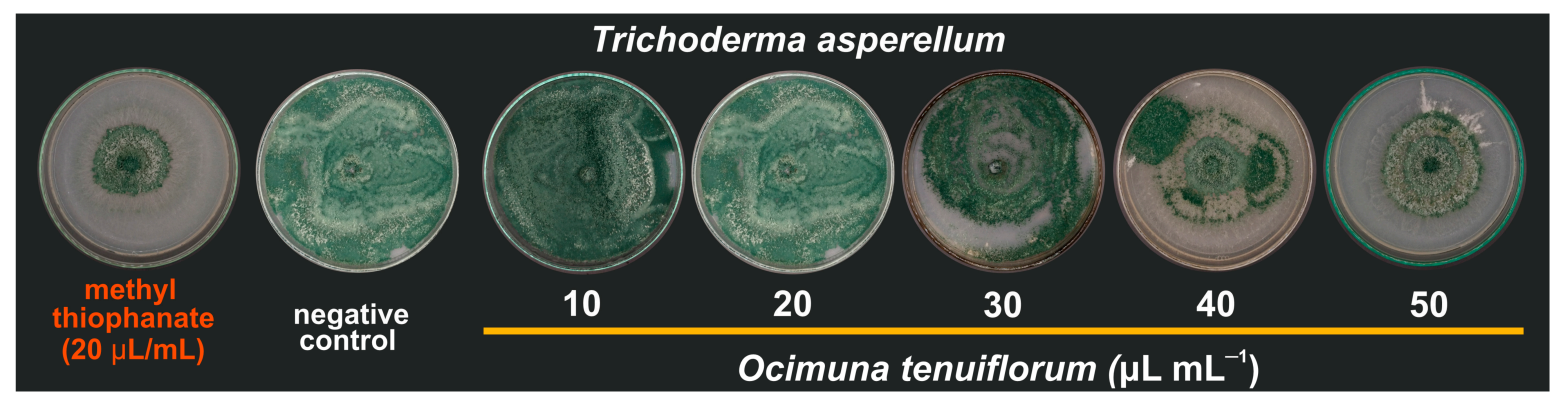
Mycelial growth of *Trichoderma asperellum* 10 days after treatment with different concentrations of *Ocimum tenuiflorum* essential oil.

Our results indicate a significant selectivity of *O. tenuiflorum* essential oil in *T. asperellum.* Ten days after exposure to different concentrations of the essential oil, mycelial growth inhibition of *T. asperellum* was not observed up to a concentration of 30 µL mL^−1^. At concentrations between 40 and 50 µL mL^−1^, there was slight inhibition of mycelial growth. In contrast, treatment with the commercial fungicide resulted in 90.58% inhibition (Table 4, Figure 7).

## 3. Discussion

Our results demonstrate that *O. tenuiflorum* essential oil represents a biorational tool for the management of *N. keratoplastica* in desert rose. Specifically, a concentration-dependent antifungal activity was observed against this pathogen, with inhibition of mycelial growth reaching approximately 50% at concentrations above 80 µL mL^−1^. However, the highest efficacy under non-phytotoxic conditions was observed at 50 µL mL^−1^ when the essential oil was applied as a preventive treatment rather than as a curative treatment. This antifungal activity is likely attributable to the major compounds identified in the essential oil, particularly eugenol and 1,8-cineole, which showed moderate affinity for the target proteins α/β-tubulin and CYP51A, as suggested by in silico analysis. In addition, other constituents such as α-copaene and dehydroaromadendrane may also contribute to the observed effects through interactions with the same molecular targets.

The chemical composition of *O. tenuiflorum* essential oil revealed that eugenol and 1,8-cineole together accounted for approximately 76% of the total content; these compounds have also been reported as major constituents in previous studies [28,33]. However, markedly different chemical profiles have also been described for this species [34], a common characteristic of essential oils due to environmental and genetic factors that influence their chemical composition and consequently, their biological activity. Thus, the antifungal activity of *O. tenuiflorum* against *N. keratoplastica,* as evidenced by mycelial growth inhibition and reduction in the Area Under the Disease Progress Curve, can be mainly attributed to these major constituents, since both eugenol and 1,8-cineole have been widely reported for their antifungal activity [30,35]. The antifungal mechanism of eugenol is associated with the disruption of membrane integrity due to its accumulation in the phospholipid bilayer, altering membrane fluidity and permeability and affecting membrane-bound proteins and enzymes [36,37]. This activity is related to its hydrophobic aromatic ring and phenolic hydroxyl group, which promote leakage of cytoplasmic contents. In addition, eugenol reduces ergosterol content, interferes with H+-ATPase activity and induces oxidative stress after short exposure periods [35,38,39], and it has been reported to exhibit antifungal activity superior to tebuconazole in some tests. In contrast, 1,8-cineole exerts its antifungal effect mainly through inhibition of ergosterol biosynthesis, reduction in cell wall rigidity, and induction of reactive oxygen species (ROS), leading to cellular damage. It also downregulated genes related to chitin synthase, ergosterol synthase, cellulase production, and glycosyl hydrolase; and shows antibiofilm activity [30,40]. Furthermore, in silico studies have identified 1,8-cineole as a bioactive ligand capable of interacting with fungal enzymes involved in metabolic pathways, including riboflavin synthase (RS), riboflavin biosynthesis protein RibD domain-containing protein (RibD), and 3,4-dihydroxy-2-butanone 4-phosphate synthase (DBPS) [41]. Additionally, compounds such as eugenol and 1,8-cineole have demonstrated antifungal activity against different fungal pathogens, with inhibitory effects varying according to the tested concentration, target species, and experimental conditions [30,42]. Therefore, it is essential to evaluate both antifungal efficacy and phytotoxicity to determine effective concentrations that provide pathogen suppression while maintaining plant safety.

On the other hand, minor compounds may also play a significant role in these biological properties, acting individually or through synergistic or antagonistic interactions. In our in silico analysis, the potential contribution of minor compounds was evaluated, and the sesquiterpenes dehydroaromadendrane and α-copaene exhibited higher binding affinities toward CYP51A and α/β-tubulin than some major constituents. CYP51A, the lanosterol 14α-demethylase essential for ergosterol biosynthesis, is inhibited by azoles through coordination with the heme iron [43]. Molecular docking results suggest that CYP51A inhibition may represent a key mechanism of antifungal action, with particular relevance of sesquiterpenes such as dehydroaromadendrane, β-bourbonene, and germacrene D, which showed strong affinity and favorable steric–hydrophobic complementarity within the catalytic site. Validation using commercial azoles confirmed the reliability of the docking protocol [44], with dehydroaromadendrane showing intermediate affinity between itraconazole and first-generation triazoles. In species of the *Fusarium solani* complex, multiple CYP51 paralogs contribute to variability in sensitivity to azole fungicides [45,46]. Similarly, α- and β-tubulin heterodimers are essential for multiple cellular processes, including maintenance of cell shape, intracellular transport, cell division, and regulation of hyphal growth [38,47,48]. This target showed consistent affinity for the terpenes α-copaene and dehydroaromadendrane. α-Copaene outperformed benzimidazole reference compounds due to its hydrophobic complementarity with the colchicine binding site, consistent with previously reported antimicrobial activity [26]. That study also reported morphological alterations and membrane damage in bacterial cells treated with α-copaene, with leakage of cellular contents confirming increased membrane permeability, as well as inhibition of biofilm formation through reduced cell surface hydrophobicity [26].

This dose–response pattern also explains the phytotoxicity observed at intermediate concentrations (50–75 µL mL^−1^), which can be attributed to the lipophilic nature of the monoterpenes and phenolic compounds present in the essential oil, allowing their incorporation into the lipid bilayer of cell membranes and thereby impairing membrane fluidity and barrier function [49,50]. Compounds such as eugenol, thymol, and carvacrol may intensify these effects by disrupting lipid–protein interactions and enhancing oxidative damage [51,52,53], such that low doses may be sublethal, whereas higher concentrations trigger severe lipid peroxidation and tissue necrosis. The greater efficacy of the preventive treatment compared with the curative treatment is consistent with this pattern, reinforcing that the effectiveness of the essential oil is strongly dependent on the timing of application. Early application may prevent critical stages of the infection process, such as spore germination and tissue penetration, which are generally more susceptible to bioactive compounds [54,55,56]. Once colonization is established, the developed mycelium may confer greater tolerance to the pathogen, making disease control more difficult [55,57]. This preventive effect may also be associated with the induction of plant defense responses triggered by essential oil components [23,27]. In this context, eugenol has been reported to induce H_2_O_2_ accumulation in tomato plants, as well as to significantly increase the activity of peroxidase, polyphenol oxidase, and phenylalanine ammonia-lyase (PAL), leading to elevated salicylic acid levels and increased expression of PR-1 proteins, a molecular marker of systemic acquired resistance (SAR) [58,59]. Similarly, Thelen et al. [60] reported that the sesquiterpene α-copaene increased in tomato plants infected with *Botrytis cinerea* but not in herbivore-damaged plants, suggesting a specific association of this compound with fungal infection responses.

The absence of inhibitory activity against *T. asperellum* demonstrates desirable biological selectivity, enabling pathogen control without compromising beneficial organisms. It is precisely this selective activity towards beneficial microorganisms [20], natural enemies [61], pollinators [24] and mainly skin cells [62] that makes essential oils promising tools for controlling microorganisms in crops. This selectivity observed against *T. asperellum* further supports the environmental safety profile of *O. tenuiflorum* essential oil, reinforcing its potential use as a biorational alternative for disease management, since it allows effective pathogen suppression while maintaining key non-target beneficial organisms.

## 4. Materials and Methods

### 4.1. Culture of the Pathogen Neocosmospora keratoplastica Isolated from Adenium obesum

The pathogen *N. keratoplastica*, previously isolated from desert rose (*A. obesum*) plants and identified based on morphological characteristics, as well as molecular and phylogenetic analyses [13], was cultured under laboratory conditions following the procedure described by Santos et al. [63]_ENREF_7. Mycelial discs (5 mm in diameter), obtained from the margins of actively growing colonies, were aseptically transferred to Petri dishes containing potato dextrose agar (PDA) and incubated at 25 ± 2 °C under a 12 h photoperiod.

### 4.2. Collection, Extraction, and Chemical Characterization of the Essential Oil of Ocimum tenuiflorum

*O. tenuiflorum* plants were collected in the municipality of Gurupi, Tocantins, Brazil (11°44′45″ S, 49°04′07″ W). The essential oil was obtained by hydrodistillation using a modified Clevenger apparatus [27,64]. Briefly, the leaves were air-dried at room temperature for five days and subsequently ground using a Wiley-type knife mill (TE-648, Tecnal, Piracicaba, Brazil). Hydrodistillation was then performed using 400 g of ground plant material and 1 L of distilled water for 2 h. The resulting extract was transferred to an amber vial, and the supernatant oil was stored at 4 °C until use in the bioassays. The chemical characterization of the essential oil was performed by gas chromatography–mass spectrometry (GC–MS) following the methodology described by Viteri et al. [65].

### 4.3. In Vitro Antifungal Activity of Ocimum tenuiflorum Essential Oil Against Neocosmospora keratoplastica Mycelial Growth

The effect of the essential oil on the mycelial growth of the pathogen was initially evaluated under laboratory conditions. Four concentrations of the essential oil (30, 40, 50, 60, 70, 80, 90 and 100 µL mL^−1^) were prepared, each tested in four replicates. The essential oil solutions were dissolved in a mixture of distilled water containing 2% dimethyl sulfoxide (DMSO) and 1% Tween-20. Subsequently, aliquots of 200 µL of each concentration were evenly spread over the surface of the culture medium in 90 mm Petri dishes using a Drigalski spreader. The plates were then inoculated with 5 mm diameter mycelial plugs placed at the center of each dish and incubated in a Biochemical Oxygen Demand (BOD) growth chamber at 25 ± 2 °C for 10 days. Mycelial growth was evaluated every two days by measuring colony diameter with a digital caliper, calculated as the mean of two orthogonal measurements taken through the center of the colony. Methyl thiophanate (20 mg mL^−1^) was used as the positive control, whereas the negative control consisted of the solution containing distilled water, Tween-20, and DMSO. The percentage of mycelial growth inhibition (MGI) was calculated based on the measurements obtained according to Krutmuang et al. [66].

### 4.4. Assessment of the Phytotoxicity of Ocimum tenuiflorum Essential Oil on Adenium obesum Seedlings

The phytotoxicity of the essential oil was evaluated in 90-day-old *A. obesum* seedlings. Concentrations of 10, 25, 50, and 75 µL mL^−1^ were evaluated, each applied to three replicate seedlings. Treatments were applied directly to the plants using manual sprayers, with 2 mL of solution applied per seedling. The negative control consisted of healthy plants treated with a solution containing distilled water, DMSO, and Tween-20, also applied to three replicate seedlings. Phytotoxicity evaluations were performed 24 h after treatment application based on the degree of injury intensity using a scale adapted from Dequech et al. [67].

### 4.5. Use of Ocimum tenuiflorum Essential Oil for the Preventive and Curative Control of Caudex Rot

Based on the results of the in vitro antifungal and in vivo phytotoxicity assays, essential oil concentrations were selected to evaluate their effects on the Area Under the Disease Progress Curve (AUDPC). For the preventive assay, 2 mL of essential oil solutions at concentrations of 10, 20, 30, 40, and 50 µL mL^−1^ were sprayed onto each seedling with three replicate seedlings per concentration, and two hours later, two mycelial discs of *N. keratoplastica* were inoculated per plant. The seedlings were maintained in a moist chamber at 25 ± 2 °C for 48 h and subsequently incubated for 10 days. The curative assay followed the same procedure but in reverse order, with the pathogen being inoculated before treatment application and the essential oil sprayed after the appearance of the first symptoms. Disease severity was assessed every 48 h over a 10-day period on both leaves and the caudex, quantified as the lesion diameter on the caudex, using a scale adapted from Santos et al. [68], and AUDPC was calculated using the AUDPC of the severity data using the formula AUDPC = Σ (y_i_ + y_i+1_)/(t_i+1_ − t_i_), where y_i_ and y_i+1_ are the severity values observed in two consecutive evaluations and t_i_ is the interval between evaluations [69,70].

### 4.6. Effect of Ocimum tenuiflorum on the Non-Target Organism Trichoderma asperellum

In the selectivity bioassay with *T. asperellum*, essential oil concentrations of 10, 20, 30, 40, and 50 µL mL^−1^ were evaluated, corresponding to those used in the preventive and curative assays. The *T. asperellum* isolate was obtained from the culture collection of the Plant Pathology Laboratory at UFT and grown on potato dextrose agar (PDA). After two days of growth, a single mycelial disc of *T. asperellum* was inoculated onto Petri dishes containing culture medium previously treated by spreading the essential oil solutions over the medium surface. The negative control consisted of distilled water containing Tween-20 and DMSO, whereas the positive control was methyl thiophanate (20 mg mL^−1^). Mycelial growth was measured every two days until the tenth day of incubation. Based on these measurements, the percentage of mycelial growth inhibition (MGI) was calculated [66].

### 4.7. Molecular Modeling, Protein Structure Preparation, and Molecular Docking

The primary sequence of the lanosterol 14α-demethylase enzyme (CYP51A) from *Fusarium keratoplasticum* (syn. *N. keratoplastica*) was retrieved from GenBank [(https://www.ncbi.nlm.nih.gov/genbank/) accessed on 23 May 2026], accession number QGR26270.1 [44]. Three-dimensional structure prediction was performed using ColabFold v1.6.2: AlphaFold2 pro alphafill using MMseqs2 public notebook [31,71]. The heme cofactor was incorporated into the predicted structure using the AlphaFill algorithm, based on structural similarity with PDB template 4UYL (*Aspergillus fumigatus* CYP51B, 58.7% identity) [72]. For heterodimer α/β-tubulin modeling, sequences were retrieved from GenBank (β-tubulin: XP_052915278.1; α-tubulin: XP_052919683.1) and processed similarly to preserve the geometry of the colchicine-binding site (CBS). Structural quality was assessed through Ramachandran plots and PROCHECK analysis via the SAVES v6.0 server [73]. Molecular docking was performed using AutoDock 4.2, with receptor and ligand preparation carried out using the Meeko toolkit [74]. EO compounds were retrieved from the PubChem database [https://pubchem.ncbi.nlm.nih.gov/; (acceded on 20 may 2026)] in SDF format. For CYP51A, the search box (22.5 × 22.5 × 22.5 Å, spacing 0.375 Å) encompassed the active site, including the heme cofactor and key residues (TYR105, PRO107, LEU108, VAL112, PHE212, THR287, MET290, ALA291, HIS294, SER295, SER361, LEU363). The search box for α/β-tubulin (15 × 15 × 15 Å) was centered on the CBS of the β-tubulin subunit, identified through structural alignment with PDB 5CA1 (69.66% identity) [75]. Positive controls included commercial antifungals: voriconazole, itraconazole, and tebuconazole (CYP51 inhibitors); carbendazim, nocodazole, and benzimidazole (tubulin inhibitors). Each ligand was subjected to 100 docking runs in triplicate. Final poses were selected based on binding affinity, inhibition constants (Ki), and structural analysis of protein–ligand interactions using PyMOL v3.1 and BIOVIA Discovery Studio Visualizer v24.1.0.23298.

### 4.8. Data Analysis

Mycelial Growth Inhibition (MGI) data were analyzed using one-way analysis of variance (one-way ANOVA) to compare the effects of the different treatments, followed by Tukey’s test for multiple comparisons (*p* < 0.0001). Data on the Area Under the Disease Progress Curve (AUDPC) obtained from the preventive and curative assays were subjected to linear regression analysis, with essential oil concentration as the independent variable and AUDPC as the dependent variable. All analyses were performed using SigmaPlot software version 12.0 (Systat Software, Chicago, IL, USA).

## 5. Conclusions

Our results demonstrated the potential of *Ocimum tenuiflorum* essential oil as a preventive treatment against *Neocosmospora keratoplastica* in desert rose, without adversely affecting beneficial fungi at moderate concentrations. These findings are particularly important because ornamental plants, especially desert rose, are commonly cultivated on balconies and verandas and therefore remain in close proximity to humans. Consequently, the use of *O. tenuiflorum* essential oil may represent a safer and more sustainable alternative to synthetic fungicides for disease management in these environments.

## Figures and Tables

**Table 1:** Chemical composition and classification of the constituents of *Ocimum tenuiflorum* essential oil obtained by GC-MS analysis.

CN	RT	Compound	RIC	RIR	(%)	SD	CG	CF
**1**	6.46	(3E)-Hexenol	871	871	0.16	0.01	OC	C_6_H_12O_
**2**	9.28	α-Pinene	929	932	0.93	0.04	MH	C_10_H_16_
**3**	11.00	Sabinene	969	969	0.88	0.04	MH	C_10_H_16_
**4**	11.20	β-Pinene	974	974	2.29	0.10	MH	C_10_H_16_
**5**	11.78	Myrcene	988	988	0.75	0.03	MH	C_10_H_16_
**6**	13.62	Sylvestrene	1027	1025	0.40	0.02	MH	C_10_H_16_
**7**	13.76	1,8-Cineole	1028	1026	29.47	1.33	OM	C_10_H_18_O
**8**	13.98	(Z)-β-Ocimene	1034	1032	5.29	0.24	MH	C_10_H_16_
**9**	15.01	γ-Terpinene	1056	1054	0.05	0.00	MH	C_10_H_16_
**10**	15.63	Sabinene hydrate	1067	1065	0.11	0.00	OM	C_10_H1_8_O
**11**	17.10	Linalool	1097	1095	0.35	0.02	OM	C_10_H1_8_O
**12**	20.47	δ-Terpineol	1165	1162	0.19	0.01	OM	C_10_H1_8_O
**13**	20.92	Terpinen-4-ol	1176	1174	0.11	0.01	OM	C_10_H1_8_O
**14**	21.65	α-Terpineol	1189	1186	0.49	0.02	OM	C_10_H1_8_O
**15**	26.27	Carvacrol	1296	1298	0.02	0.00	OM	C_10_H1_4_O
**16**	28.98	Eugenol	1353	1356	47.04	2.12	OM	C_10_H_12_O_2_
**17**	29.84	α-Copaene	1373	1374	0.09	0.00	SH	C_15_H_24_
**18**	30.19	β-Bourbonene	1380	1387	0.05	0.00	SH	C_15_H_24_
**19**	30.46	β-Elemene	1387	1389	0.19	0.01	SH	C_15_H_24_
**20**	31.73	(E)-Caryophyllene	1416	1417	2.69	0.12	SH	C_15_H_24_
**21**	33.25	α-Humulene	1452	1452	0.36	0.02	SH	C_15_H_24_
**22**	33.43	dehydroaromadendrane	1458	1460	0.05	0.00	SH	C_15_H_24_
**23**	34.33	Germacrene D	1478	1480	0.77	0.03	SH	C_15_H_24_
**24**	34.66	β-Selinene	1487	1489	4.23	0.19	SH	C_15_H_24_
**25**	34.95	γ-Amorphene	1492	1495	1.25	0.06	SH	C_15_H_24_
**26**	35.89	7-epi-α-Selinene	1519	1520	0.29	0.01	SH	C_15_H_24_
**27**	38.42	Caryophyllene oxide	1579	1582	0.08	0.00	OS	C_15_H_24_O
Monoterpene hydrocarbons (MH)					10.59			
Oxygenated monoterpenes (OM)					77.78			
Sesquiterpene hydrocarbons (SH)					9.97			
Oxygenated sesquiterpene (OS)					0.08			
Other compounds (OC)					0.16			
Total identified					98.58			

CN: compound number, assigned according to their elution order; RT: retention time; RIC: calculated retention indices; RIR: literature retention index; SD: Standard Deviation; %: relative abundance; CG: Compound group; CF: Chemical formula.

**Table 2:** Inhibitory Effect of *Ocimum tenuiflorum* Essential Oil on the Mycelial Growth of *Neocosmospora keratoplastica*.

Treatments (µL mL^−1^)	Mycelial Growth Over Time (mm ± SE)					MGI(%)
	2 Days	4 Days	6 Days	8 Days	10 Days	
DMSO and Tween 20 solution	34.5 ± 0.63	52.51 ± 0.55	90 ± 0	90 ± 0	90 ± 0	0.00 ^b^
Methyl thiophanate *	31.73 ± 0.29	49.55 ± 1.11	67.92 ± 1.26	89.82 ± 0.11	90 ± 0	7.68 ^b^
30	32.5 ± 0.65	50.36 ± 0.58	90 ± 0	90 ± 0	90 ± 0	2 ^b^
40	34.43 ± 0.11	44.3 ± 0.5	68.18 ± 1.28	90 ± 0	90 ± 0	8 ^b^
50	27.3 ± 1.7	38.96 ± 1.61	75.37 ± 0.91	77.47 ± 0.73	78.41 ± 0.55	18 ^b^
60	17.62 ± 0.39	28.49 ± 0.33	45 ± 0.47	50.63 ± 0.12	68.67 ± 0.34	42 ^a^
70	16.68 ± 0.55	25.21 ± 1.42	38.88 ± 1.64	45.15 ± 0.15	63.41 ± 1.46	48 ^a^
80	16.2 ± 0.21	23.11 ± 0.43	35.89 ± 0.24	40.14 ± 0.28	57.41 ± 0.49	52 ^a^
90	15.41 ± 0.1	21.96 ± 0.11	31.63 ± 0.32	35.61 ± 0.12	53.15 ± 0.27	56 ^a^
100	13.74 ± 0.33	19.99 ± 0.34	30.34 ± 0.38	32.23 ± 0.26	50.86 ± 0.12	59 ^a^

Different lowercase letters in the MGI column indicate statistically significant differences among treatments according to Tukey’s test (*p* < 0.0001); * 20 µL mL^−1^; MGI: Mycelial Growth Inhibition.

**Table 3:** Heatmap of estimated binding energies (ΔG) obtained by molecular docking for 27 essential oil ligands and synthetic fungicides against the target proteins CYP51A and α/β tubulin.

Ligands	CYP51A (kcal/mol)	α/β-Tubulin (kcal/mol)
(3E)-Hexenol	−3.89	−3.17
α-Pinene	−6.28	−5.51
Sabinene	−6.18	−5.31
β-Pinene	−6.27	−5.52
Myrcene	−5.41	−4.55
Sylvestrene	−5.98	−5.15
1,8-Cineole	−5.88	−5.54
(Z)-β-Ocimene	−5.66	−4.77
γ-Terpinene	−5.97	−5.16
Sabinene hydrate	−6.3	−5.65
Linalool	−5.76	−4.74
δ-Terpineol	−6.43	−5.49
Terpinen−4-ol	−6.22	−5.41
α-Terpineol	−6.51	−5.36
Carvacrol	−5.67	−4.83
Eugenol	−5.10	−4.16
α-Copaene	−8.14	−7.39
β-Bourbonene	−8.61	−7.18
β-Elemene	−7.7	−6.73
(E)-Caryophyllene	−8.41	−6.93
α-Humulene	−8.01	−6.91
dehydro-Aromadendrane	−8.69	−7.25
Germacrene D	−8.59	−6.68
β-Selinene	−8.27	−6.29
γ-Amorphene	−8.26	−7.16
7-epi-α-Selinene	−8.13	−6.86
Caryophyllene oxide	−8.68	−6.83
Itraconazole ^a^	−10.1	
Voraconazole ^a^	−6.81	
Tebuconazole ^a^	−6.87	
Benzimidazole ^a^		−3.86
Nocodazole ^a^		2.75

^a^ synthetic fungicide.

**Table 4:** Effect of *Ocimum tenuiflorum* essential oil on the inhibition of mycelial growth of *Trichoderma asperellum*.

Treatments (µL mL^−1^)	Mycelial Growth Over Time (mm ± SE)					MGI (%)
	2 Day	4 Day	6 Day	8 Day	10 Day	
Negative Control	90 ± 0	90 ± 0	90 ± 0	90 ± 0	90 ± 0	0.00 ^a^
Methyl thiophanate *	0 ± 0	10.6 ± 0.51	10.6 ± 0.51	10.6 ± 0.51	10.6 ± 0.51	90.58 ^b^
10–40	90 ± 0	90 ± 0	90 ± 0	90 ± 0	90 ± 0	0 ^a^
50	59.33 ± 3.91	90 ± 0	90 ± 0	90 ± 0	90 ± 0	7 ^a^

Different lowercase letters in the MGI column indicate statistically significant differences among treatments according to Tukey’s test (*p* < 0.0001); * 20 µL mL^−1^.

## Data Availability

The original contributions presented in this study are included in the article/Supplementary Material. Further inquiries can be directed to the corresponding author.

## Supplementary Materials

The following supporting information can be downloaded at: https://www.mdpi.com/article/10.3390/plants15152332/s1, Figure S1: Three-dimensional structural model of the CYP51A protein colored by pLDDT values overview of the model, highlighting the predominance of α-helices in the catalytic core (A); Detail of the active site, showing the heme group accommodation region (B); Structural validation analyses of the CYP51A model: (C) Ramachandran plot; (D) PAE matrix; Figure S2: Structural model of the protein with local confidence mapping (pLDDT) and active site organization at the interface between the α (A) and β subunits (B); Structural validation analyses of the α/β heterodimeric model: (C) Ramachandran plot; (D) PAE matrix.
