# Supplementary material for: Ocimum tenuiflorum Essential Oil as a Safe Tool to Prevent Neocosmospora keratoplastica Fungus in Adenium obesum Ornamental Plant
Source: Plants (Basel). 2026 Jul 29;15(15):2332. doi: 10.3390/plants15152332 (PMC13467860; doi:10.3390/plants15152332)

**Figure S1**. Three-dimensional structural model of the CYP51A protein colored by pLDDT values overview of the model, highlighting the predominance of $\alpha$-helices in the catalytic core (A); Detail of the active site, showing the heme group accommodation region (B); Structural validation analyses of the CYP51A model: (C) Ramachandran plot; (D) PAE matrix.

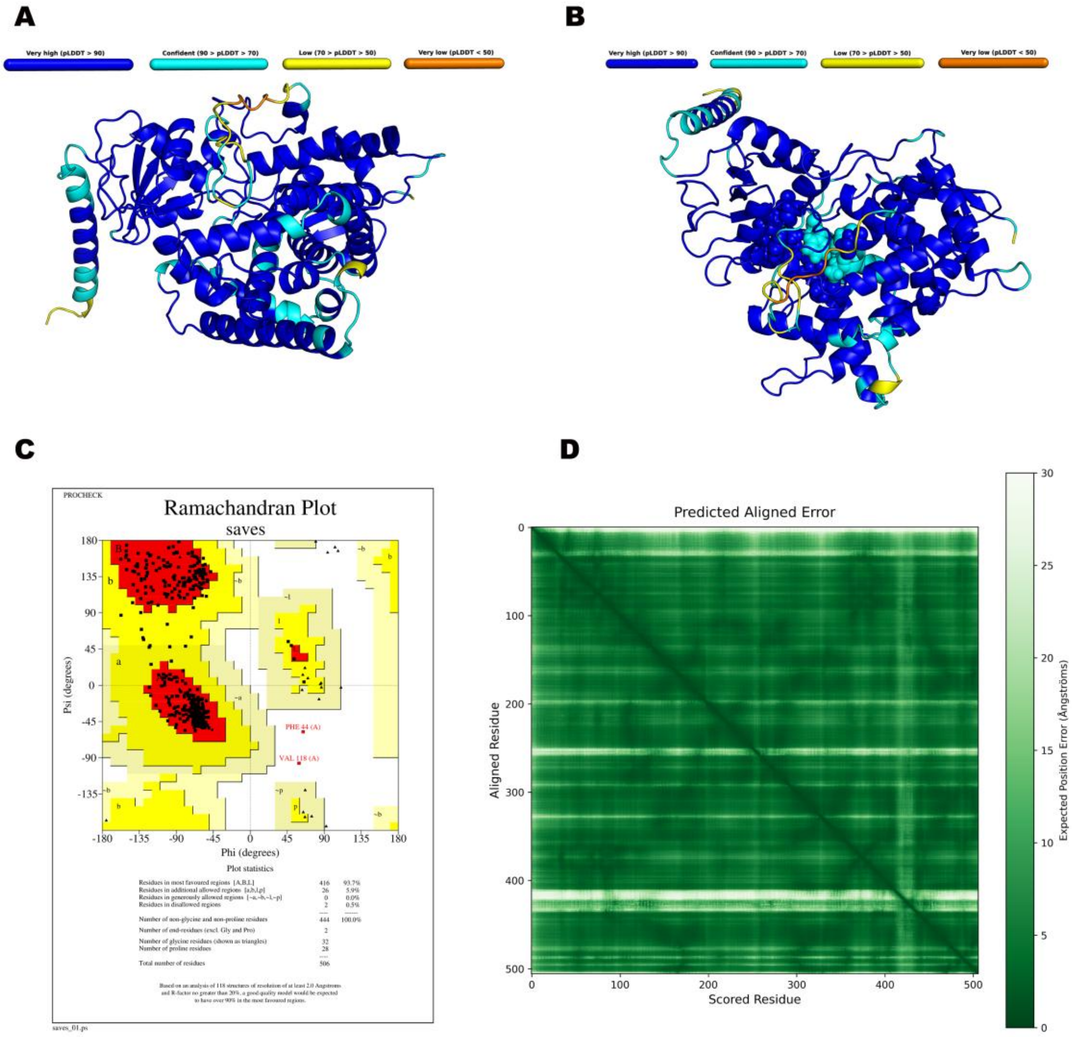

**Figure S2**. Structural model of the protein with local confidence mapping (pLDDT) and active site organization at the interface between the $\alpha$ (A) and $\beta$ subunits (B); Structural validation analyses of the $\alpha/\beta$ heterodimeric model: (C) Ramachandran plot; (D) PAE matrix.

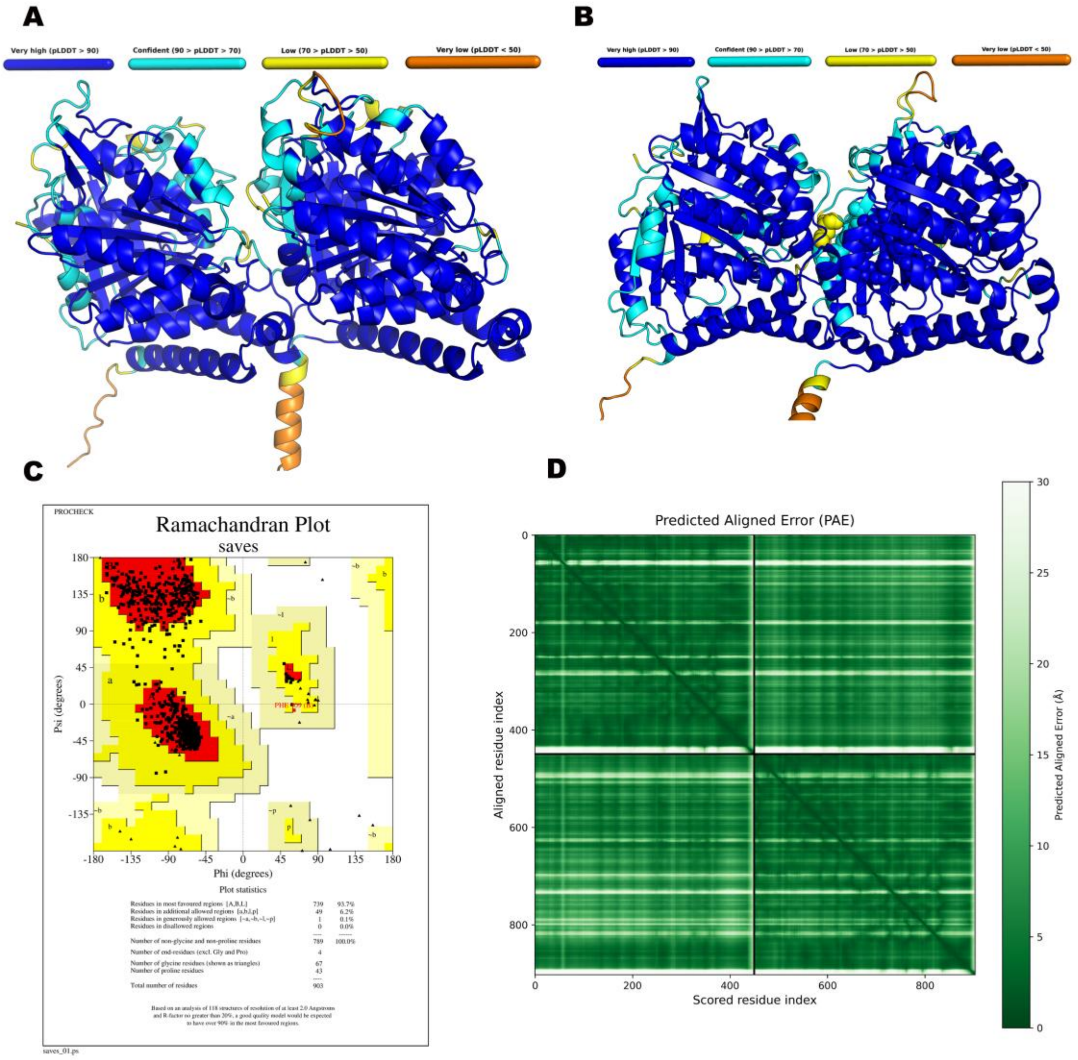

Supplement: Supplementary file 1 [file plants-15-02332-s001.zip › plants-4450190-supplementary.pdf]
